# Own Song Selectivity in the Songbird Auditory Pathway: Suppression by Norepinephrine

**DOI:** 10.1371/journal.pone.0020131

**Published:** 2011-05-23

**Authors:** Colline Poirier, Tiny Boumans, Michiel Vellema, Geert De Groof, Thierry D. Charlier, Marleen Verhoye, Annemie Van der Linden, Jacques Balthazart

**Affiliations:** 1 Bio-Imaging Lab, Department of Biomedical Sciences, University of Antwerp, Antwerp, Belgium; 2 Department of Behavioral Neurobiology, Max Planck Institute for Ornithology, Seewiesen, Germany; 3 Research Unit in Behavioral Neuroendocrinology, GIGA Neurosciences, University of Liège, Liège, Belgium; Claremont Colleges, United States of America

## Abstract

**Background:**

Like human speech, birdsong is a learned behavior that supports species and individual recognition. Norepinephrine is a catecholamine suspected to play a role in song learning. The goal of this study was to investigate the role of norepinephrine in bird's own song selectivity, a property thought to be important for auditory feedback processes required for song learning and maintenance.

**Methodology/Principal Findings:**

Using functional magnetic resonance imaging, we show that injection of DSP-4, a specific noradrenergic toxin, unmasks own song selectivity in the dorsal part of NCM, a secondary auditory region.

**Conclusions/Significance:**

The level of norepinephrine throughout the telencephalon is known to be high in alert birds and low in sleeping birds. Our results suggest that norepinephrine activity can be further decreased, giving rise to a strong own song selective signal in dorsal NCM. This latent own song selective signal, which is only revealed under conditions of very low noradrenergic activity, might play a role in the auditory feedback and/or the integration of this feedback with the motor circuitry for vocal learning and maintenance.

## Introduction

The role of norepinephrine (NE) in the neurobiology of birdsong has recently been the focus of multiple studies but still remains poorly understood. Previous neuroanatomical and neurochemical studies have identified a dense noradrenergic innervation of the song control and auditory brain regions of songbirds [1]–[5]. The presence of high concentrations of NE and NE receptors in the song control and auditory nuclei, associated with the observation that the development of the noradrenergic innervation closely parallels song learning, have led to the hypothesis that NE might be involved in the control of song production, perception and learning (for a review, see [6]).

The songbird brain is able to discriminate between the bird's own song (BOS) and other conspecific (CON) songs [7]–[9]. Since song is a learned behavior, the development of BOS selectivity necessarily involves experience-dependent mechanisms, and brain regions sensitive to self-generated vocalizations could mediate the auditory feedback critical for song learning and song maintenance. Electrophysiological evidence indicates that NE can suppress BOS responsiveness in song control regions [10], [11]. The main goal of the present study was to further investigate the role of NE on BOS selectivity.

The selective noradrenergic neurotoxin DSP-4 causes substantial and long-lasting depletion of NE inputs in the mammalian and avian telencephalon that derive from the locus coeruleus and the nucleus subcoeruleus ventralis while leaving noradrenergic inputs to the hypothalamus originating from other noradrenergic cell groups relatively unaffected (see [12]–[15] for discussion). The mechanisms controlling this differential impact of the drug have not been identified to our knowledge. DSP-4 has been widely used to lower brain NE in order to investigate the functions of the central noradrenergic system in birds [14], [16]–[22]. Using functional Magnetic Resonance Imaging (fMRI), we assessed how DSP-4-induced noradrenergic denervation affects BOS selectivity in the telencephalic auditory and song control regions of anesthetized male zebra finches, namely Field L, NCM, CMM, CML, HVC, RA, LMAN and area X (see material and methods section for the meaning of the abbreviations).

## Results

### Effects of DSP-4 on noradrenergic cells

**Figure 1:**
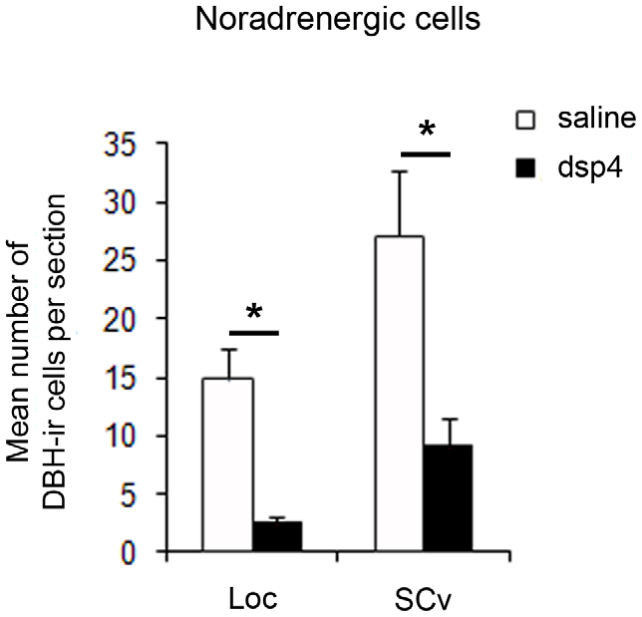
DSP-4 effect on noradrenergic cells. Mean number of DBH-immunoreactive (DBH-ir) cells per section in the locus coeruleus (Loc) and the nucleus subcoeruleus ventralis (SCv) of the saline (white bars) and DSP-4 (black bars) groups. The error bars correspond to standard errors across subjects.

The effect of the drug treatment was assessed by counting the number of cells in the locus coeruleus and the nucleus subcoeruleus ventralis expressing dopamine ß-hydroxylase (DBH), the rate-limiting enzyme in NE synthesis. The immunohistochemical staining revealed a prominent decrease in the number of DBH-immunoreactive cells in the two nuclei (locus coeruleus: t = 5.684, df = 5, p = 0.003; nucleus subcoeruleus ventralis: t = 2.958, df = 6, p = 0.025) in the DSP-4 group as compared to the saline group (Fig. 1). These decreases represented an 88% loss of locus coeruleus cells and a 66% loss of ventral sub-coeruleus immunoreactive cells.

### Effects of DSP-4 on BOS selectivity

**Figure 2:**
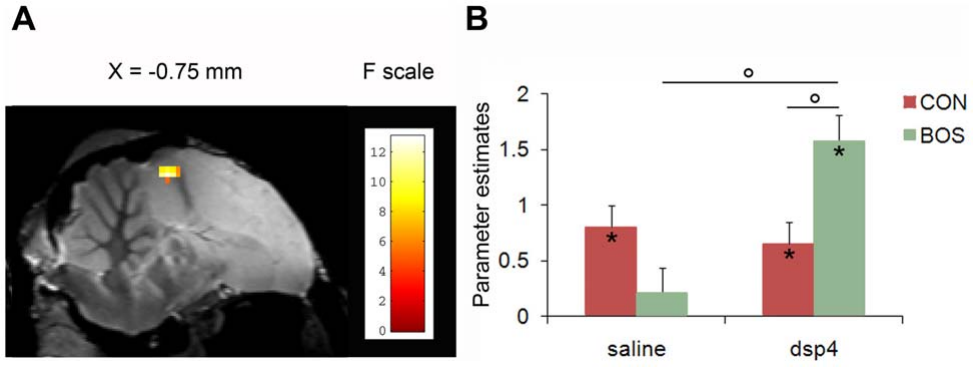
DSP-4 effect on BOS selectivity. **A** Statistical map of voxels displaying a significant difference in BOS selectivity between groups in the song control and auditory regions (F-test). F values are color coded according to the scale displayed on the right side of the figure. Statistical results are superimposed to an anatomical sagittal image coming from the MRI zebra finch atlas. The position of the slice along the X (left/right) axis is indicated above the map (the - sign indicates that slices and statistical results are from the left hemisphere). **B** Estimates of the relative response amplitude (derived from the Restricted Maximum Likelihood algorithm, expressed in non-dimensional units) elicited by CON (in red) and BOS (in green) stimuli in the cluster illustrated in panel A (the values have been extracted from the voxel with the maximum F value). The zero level corresponds to the estimated mean activation during rest periods. The error bars correspond to standard errors across subjects. Stars indicate statistically significant differences between stimuli vs. rest. Circles indicate statistical significance of comparisons between stimuli (paired t-tests) and between groups (unpaired t-tests).

Three song stimuli were used in the fMRI experiment: the BOS, a familiar conspecific song (CON) and a heterospecific song (HET). BOS selectivity was assessed in each experimental group by comparing the neural activation induced by the perception of the BOS and the perception of the CON ([BOS minus CON]). Potential DSP-4 effects were measured by comparing BOS selectivity between the two experimental groups ([BOS minus CON]_dsp-4_ vs. [BOS minus CON]_saline_). This analysis revealed a significant difference in a cluster located in the dorsal part of the left NCM extending to dorsal Field L (F_max_ i.e. voxel presenting the maximal F value among all significant voxels of the cluster  = 13.02, p = 0.008) (Fig. 2). A similar non-significant trend was found in the right dorsal NCM/Field L region. The presence of this trend argues against a lateralization of the effect but does not allow excluding it. Post-hoc analyses of the significant cluster in left NCM/Field L indicated a significantly higher activation induced by BOS than by CON in the DSP-4 group (t_max_ i.e. voxel presenting the maximal t value among all significant voxels of the cluster  = 3.52, p = 0.003). Spin-echo fMRI, as implemented in the present study, can detect statistically significant differential activations triggered by different stimuli at the group level but not at the individual level [23]. Nevertheless, it is noteworthy that BOS-induced activity in the cluster was higher than CON-induced activity in all DSP-4 treated birds (N = 6). This BOS selectivity was substantiated by the comparison [BOS minus HET] (t_max_ = 3.7; p = 0.002), with HET being considered as a natural complex auditory control stimulus. This significant BOS selectivity of dorsal NCM/Field L was absent in the saline group ([BOS minus CON]: t_max_ = 0.03; p = 0.71). A previous study using the same stimuli and the same methodology in a group of non-treated birds also failed to detect any BOS selectivity in the same region [24]. Finally, the BOS selectivity specifically observed in the DSP-4 group was found to result from a significantly greater neural activity induced by BOS in the DSP-4 group as compared to the saline group (t_max_ = 2.64; p = 0.028). We did not find any significant DSP-4 effects on BOS selectivity in the telencephalic song control nuclei. Nevertheless, a non-significant trend was found in the border of right area X, where BOS selective responses were already found in our previous study [24]. This non-significant effect consisted of a trend for increased BOS selective responses in the saline group compared to the DSP-4 group. While the neural response induced by each stimulus in the DSP-4 group seemed higher compared to those in the saline group, the effect was greater for CON and HET than for BOS, resulting in a lack of BOS selectivity in the DSP-4 group. No consistent trend was found in HVC or HVC shelf.

### Effects of DSP-4 on CON selectivity

**Figure 3:**
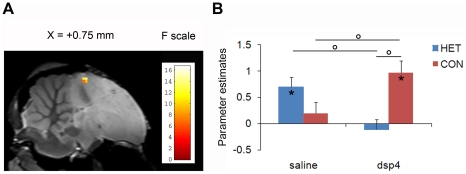
DSP-4 effect on CON selectivity. **A** Statistical map of voxels displaying a significant difference in CON selectivity between groups in the song control and auditory regions (F-test). The position of the slice along the X (left/right) axis is indicated above the map (the + sign indicates that the slice and statistical results are from the right hemisphere). **B** Estimates of the relative response amplitude (expressed in non-dimensional, arbitrary units) elicited by HET (in blue) and CON (in red) stimuli in the cluster illustrated in panel A (the values have been extracted from the voxel with the maximum F value). The zero level corresponds to the estimated mean activation during rest periods. The error bars correspond to standard errors across subjects. Stars indicate statistically significant differences between stimuli vs. rest. Circles indicate statistical significance of comparisons between stimuli (paired t-tests) and between groups (unpaired t-tests).

The comparison of the neural activation induced by the perception of the CON and the perception of the HET also allowed us to assess CON selectivity ([CON minus HET]). Potential DSP-4 effects were similarly measured by comparing CON selectivity between the two experimental groups ([CON minus HET]_dsp-4_ vs. [CON minus HET]_saline_). This analysis revealed a significant difference in a cluster located in the right CMM (F_max_ = 14.40, p =  0.005) (Fig. 3). No similar trend was found in the left CMM, suggesting a potential lateralization of this effect. However, due to the small number of birds included in this study, this result should be considered with caution and future studies should be performed to draw firm conclusions. Post-hoc analyses in the right CMM cluster indicated a significant CON selectivity in the DSP-4 group (t_max_ = 3.44; p = 0.002). Individual analyses revealed a CON-induced activity higher than HET-induced activity in five DSP-4 treated birds (out of six). Such a CON selectivity was absent in the saline group (CON minus HET: t_max_ = −1.54; p = 0.62) as well as in the group of non-treated birds previously scanned while exposed to the same stimuli [24]. The CON selectivity only present in the DSP-4 group resulted from a significantly greater neural activity induced by CON (t_max_ = 3.31; p = 0.002) and a weaker neural activity induced by HET (t_max_ = 2.81; p = 0.009) in this group, compared to the saline group. No significant difference was found in the telencephalic song control nuclei.

## Discussion

The goal of the present study was to investigate the role of NE on BOS selectivity. To manipulate NE level in the zebra finch brain, we systemically injected birds with DSP-4, a neurotoxin known to induce a retrograde degeneration of the two main noradrenergic cell groups innervating the telencephalon, the locus coeruleus and nucleus subcoeruleus ventralis [15], [20]. The effectiveness of the pharmacological treatment was confirmed by immunohistochemical staining for DBH, the rate-limiting enzyme for NE synthesis: the number of DBH immunoreactive cells in the locus coeruleus and the nucleus subcoeruleus ventralis were found to be drastically reduced in the DSP-4 treated birds compared to a control group of saline treated birds (locus coeruleus: −88%; nucleus subcoeruleus ventralis: - 66%). This neurochemical depletion was associated with significant changes in auditory processing.

### Specificity of DSP-4 effects

The present fMRI study demonstrated that the DSP-4 treatment induced a marked BOS selectivity in dorsal NCM, a secondary auditory region and CON selectivity in CMM, another secondary auditory region. These auditory regions of the telencephalon are known to contain NE receptors like many nuclei of the song control system [25]–[27]. These auditory regions also receive a dense noradrenergic innervation [4]. Previous work in zebra finches specifically demonstrated that DSP-4 markedly depletes NE levels in multiple telencephalic brain sites [17] and that DSP-4-induced depletions specifically affect NE without changing the concentration of other amines [14], [17], [28], [29]. The selective auditory responses observed here in the DSP-4 group but not in the control group are thus likely to be a consequence of the NE depletion induced by the DSP-4 treatment.

### Effects on BOS selectivity

In zebra finches, BOS selective responses are present in the song control system of sedated, anaesthetized and sleeping birds, when NE level is low [7]–[9], [30]. These BOS selective responses are suspected to play an off-line role in song learning and maintenance [31]. However, BOS selective responses are suppressed by arousal, when NE level is high [11], [32]. This state dependence is mediated by the noradrenergic system: injection of a high-dose of NE in the nucleus interface of the nidopallium (NIf), a major auditory input to the song control nucleus HVC, suppresses BOS responsiveness in HVC while injection of adrenergic antagonists in NIf blocks the arousal-mediated suppression of BOS responsiveness in HVC [11]. Injection of NE in HVC was also found to suppress BOS responsiveness in RA, a song control nucleus receiving dense projections from HVC [10]. These results indicate that a high level of NE during arousal is responsible for the lack of BOS responsiveness in the song control system of alert zebra finches. The present study shows that DSP-4 injection, decreasing the level of NE, has additional effects on BOS selectivity. A significant effect was found in a brain region encompassing the dorsal part of NCM, and consisted in a specific increase of BOS responsiveness, giving rise to a strong BOS selective signal in this auditory region. The functional significance of these latent BOS responsiveness and BOS selectivity that are only revealed under condition of very low noradrenergic activity remains unclear at present. Birdsong is learned and maintained by a trial and error process, in which the bird uses auditory feedback to actively match its vocal output to a memorized model (the tutor song). The birdsong brain thus needs to evaluate the vocal output (error detection) and to drive adaptive changes in song to decrease the difference between auditory feedback and memorized tutor song (error correction). BOS-selective responses are thought to support the auditory feedback and/or the integration of this feed-back with the motor circuitry for vocal learning and maintenance [33], [34]. The BOS selective signal revealed in the present experiment under condition of very low noradrenergic activity might thus play a role in these processes. Interestingly, this signal was found localized in NCM, a brain region where a memory trace of the tutor song is suspected to be stored [35], [36]. Finally, feedback-sensitive neurons have been recently identified in other forebrain auditory regions: in these regions, BOS responsiveness was found to increase when the birds were singing or hearing a perturbed song [37], [38]. Even if a comprehensive picture of the neural mechanisms supporting auditory-vocal integration still needs to emerge, these findings point to a crucial role of the ascending auditory pathway in vocal error detection.

As mentioned earlier, several electro-physiological studies have demonstrated NE effects on BOS selective responses in song control nuclei [10], [11]. Based on this literature, one might have expected DSP-4 effects in the song control regions to be observed in the present study. We did not find significant effects in these regions. However, this absence of significant results should be interpreted cautiously. The fact that the fMRI signal is usually lower in the song control nuclei compared to the auditory regions (this study and [24]) combined with the limited number of subjects involved in the present study drastically reduced the statistical power of the analysis in these regions. Consistent with the interpretation of a lack of DSP-4 effects due to a too low statistical power, we did find a non-significant effect in the border of area X. In line with BOS selective responses found in the same region in our previous study [24], we found a trend for BOS selective responses in the saline treated birds but not in the DSP-4 treated birds. Interestingly, data suggest that DSP4 injection resulted in an increase of the neural activity induced by the three stimuli but to a lesser extent by BOS than CON and HET stimuli. If this non-significant effect is confirmed by further experiments, it will indicate a qualitative difference of DSP-4 effects between the auditory and song control regions. In our previous experiment [24], we also found BOS selective responses in the HVC shelf, generalizing to large population of neurons what was previously found with electro-physiology in a small number of neurons [8]. In the present study, we did find BOS selective responses in some birds of each group but results were too variable to give rise to any consistent effect within and between groups. In addition to the limited statistical power of the study that can explain this absence of results, it should be noted that because HVC is situated at the top of the songbird brain, the signal is often contaminated by MRI artifacts due to brain tissues/air interface. These artifacts make particularly challenging to measure a robust and reliable fMRI signal in this region.

### Effects on CON selectivity

DSP-4 injection also induced CON selectivity in CMM, another secondary auditory region. This selective signal was due to a higher neural activity induced by CON and a lower activity induced by HET in the DSP-4 treated birds, compared to the saline-treated birds. Previous studies in awake birds suggest an important role of NE in selective attention to sexually relevant stimuli (e.g. [18], [21], [22], [39]). For instance, Lynch and Ball [21] found that DSP-4 injection in awake female canaries induces a decrease of neuronal activity (measured by ZENK expression) triggered by male song perception in NCM and CMM. These results might seem in opposition with the increase of CON-related neural activity observed in the present study in DSP-4 treated birds. It should however be noted that, in addition to the species and sex difference, our birds were anesthetized, preventing us to draw any conclusion in terms of attention. It has also been suggested that NE plays a different role within and outside the reproductive context [40]. For instance, DSP-4 administration to male zebra finches increases ZENK expression in area X induced by female-directed singing motor behavior but not by (non-sexual) undirected singing [20]. It should be noted that only undirected songs were tested in our study.

Like BOS selectivity observed in dorsal NCM, CON selectivity present in CMM might play a role in song learning and maintenance. Song learning in juvenile birds is biased in favor of copying conspecific songs rather than heterospecific songs. The CON selective signal in CMM might play a role in learning to produce a zebra finch song while the BOS selective signal in dorsal NCM could be involved in copying one specific zebra finch song, the tutor song.

### Conclusion

The present study illustrates the usefulness of fMRI to investigate suspected effects whose precise location is not *a priori* known. Specifically, we show here that brain NE depletions affect in a very specific manner neural activity related to song perception in CMM and dorsal NCM. The neurochemical mechanisms mediating these effects could now be analyzed by imaging birds exposed to a variety of specific agonists or antagonists of the noradrenergic receptor sub-types.

## Materials and Methods

### Ethics Statement

Experimental procedures were in agreement with the Belgian laws on the protection and welfare of animals and were approved by the ethical committee of the University of Antwerp, Belgium (Permit number: 2007-12).

### Subjects

Sixteen adult male zebra finches (*Taeniopygia guttata*) purchased from local suppliers were used in this experiment. Birds were housed in aviaries under a 12 h light: 12 h dark photoperiod and had access to food and water *ad libitum* throughout the experiment.

### Pharmacological treatments

Birds were first pre-treated with an i.p. injection of zimelidine hydrochloride (20 mg/kg; Sigma), a serotonin reuptake blocker used to protect serotonergic neurons from DSP-4 (see 14). One hour later, birds were injected i.p. with DSP-4 (N-(2-chloroethyl)-N-ethyl-2-bromobenzylamine hydrochloride, 50 mg/kg, Sigma) (n = 8) or saline solution (0.9%) (n = 8).

### Stimuli and fMRI experimental protocol

After seven days of recovery from these pharmacological treatments in individual cages, all birds were imaged in an MRI scanner while being exposed to three different acoustic playbacks. The full description of the acoustic stimuli and the experimental protocol can be found in one of our previous papers [24]. Briefly, the protocol consisted of a block design alternating auditory stimulation periods with resting periods. Three different auditory stimuli were presented to each bird: a heterospecific song (canary or starling song, HET), a conspecific song (undirected song from a cage mate, CON) and the bird's own song (undirected song, BOS). The duration of the stimuli was 16 s and their intensity was matched in term of Root-Mean-Square. Each stimulus was presented 25 times, resulting in the acquisition of 50 MRI scans per stimulus and per subject (2 scans per presentation). The presentation order of the three stimuli was randomized within and between subjects.

During the experiment, birds were anesthetized with isoflurane (2%). Body temperature was continuously monitored with a cloacal temperature probe and maintained at 40 °C by a feedback controlled heating system (SA-Instruments, USA). The 7T MR scanner and the RF coils used for the experiment are described in Boumans et al. [41]. Anatomical 3D images required for localization of the functional data were obtained for each bird using a T_2_-weighted Fast Spin Echo sequence (TE/TR: 60/2000 ms, voxel size: 0.0625×0.0625×0.0625 mm^3^). Blood oxygen level dependent (BOLD) fMRI data were acquired with a T_2_-weighted Fast Spin Echo sequence (TE/TR: 60/2000 ms). Fifteen continuous sagittal slices of 0.75 mm thickness covering nearly the entire brain were acquired within 8 s. Voxel size was 0.25×0.25×0.75 mm^3^.

### Functional MRI data processing and statistical analyses

Functional images were realigned, normalized to the zebra finch brain MRI atlas [42], and smoothed with a 0.5-mm width Gaussian kernel (for more details, see 24). Statistical voxel-based analyses were carried out using SPM5 (http://www.fil.ion.ucl.ac.uk/spm/). Data were modeled as a box-car and filtered with a high-pass filter of 360 s. Model parameters were then estimated using a classical Restricted Maximum Likelihood algorithm and a mixed-effect analysis was performed.

We first computed the mean effect of each stimulus (as compared to the rest period) in each voxel, for each subject of each group. In a second step, a group analysis was performed on the effects identified by the previous analysis. The individual analyses revealed a BOLD response triggered by the auditory stimuli in the bilateral Field L (equivalent of the primary auditory cortex in mammals) in 7 of the 8 saline treated birds and 6 out the 8 DSP-4 treated birds (Figure S1). This success rate is similar to the one obtained in our previous spin-echo fMRI experiments [23], [24]. The group analysis was thus only performed on these birds. The statistical analysis was restricted to *a priori* defined regions of interest (ROI), first the song control nuclei: HVC, used as a proper name [43], the nucleus robustus of the arcopallium (RA), area X and the lateral magnocellular nucleus of the anterior nidopallium (LMAN), and secondly the auditory regions: Field L, the caudal medial nidopallium (NCM), the medial part of the caudal mesopallium (CMM), and the lateral part of the caudal mesopallium (CML). Because they were too small to be sampled reliably in one sagittal slice, the dorso-lateral nucleus of the medial thalamus, the nucleus ovoidalis and NIf were not investigated. Delineations of the nuclei on the zebra finch atlas [42] were used to define the ROI. Note that in the atlas, the dark region in the auditory forebrain is interpreted as the whole Field L and not only as the subdivision L2 due to its size, its position and its shape (see three-dimensional rendering in [42]), and the fact that a darker sub-region likely to be L2 is visible in the most frontal part of the nucleus (see fig. 3 in the present manuscript). NCM, CMM and CML are not visible on the atlas. NCM was thus defined as the region located between Field L and the cerebellum and the caudal mesopallium as the region dorso-frontal to Field L but ventral to the lateral ventricle. The limit between the medial and the caudal part of the mesopallium was set at 1 mm lateral to the midline.

The aim of this study was to investigate the role of NE on BOS selectivity (as defined by BOS- minus CON-related activity) and on CON selectivity (as defined by CON- minus HET-related activity). We thus first identified voxels that displayed a differential response to the stimuli as a function of experimental treatments by the presence of a significant interaction “stimulus×group” in the 3×2 ANOVA (within factors: three stimuli; between factors: 2 groups) in the predefined ROI. In a second step, we focused exclusively on these voxels and investigated the nature of this general interaction by testing more specifically for potential interaction between groups and BOS selectivity (within factors: BOS and CON stimuli; between factors: groups) or CON selectivity (within factors: CON and HET stimuli; between factors: groups). Post-hoc t-tests were then performed (paired t-tests for intra-group comparisons; unpaired t-tests for inter-group comparisons) only on the voxels found to be significant in the interactions. Because statistical tests were performed on a voxel basis resulting in numerous tests, p values were adjusted to the number of independent tests performed. This was done using the Family Wise Error method. This method uses the Random Field Theory to calculate the number of independent tests, taking into account the number of voxels but also the amount of auto-correlation among data.

### Brain fixation and immunohistochemistry

Birds were sacrificed one day after the fMRI experiment by decapitation. Brains were dissected out of the skull and fixed in acrolein solution (Fluka Biochemika; 5% in Phosphate Buffered Saline) for 2.5 h, washed twice for 30 min in Phosphate Buffered Saline, cryoprotected overnight in 30% sucrose solution, frozen on dry ice and stored at −80°C.

Brains were then cut in the coronal plane with a cryostat in four series of 50 µm thick sections. Two alternate series (one section every 100 µm) were stained by immunohistochemistry for DBH, the rate-limiting enzyme in NE synthesis, with a commercially available rabbit polyclonal DBH antibody (Immunostar Inc., Hudson WI, USA) following a standard avidin-biotin protocol. Briefly, free-floating sections were rinsed in 0.1% sodium borohydrate in Tris Buffered Saline, blocked in 5% normal goat serum and incubated overnight in the primary antibody at 1∶6000 in Tris Buffered Saline containing 0.1% triton X100. The following day, sections were incubated with a biotinylated goat anti-rabbit secondary antibody, and processed through the avidin-biotin Vectastain Elite procedure (peroxidase) as described by the manufacturer (Vector Labs, Burligame CA, USA). The enzymatic activity was then visualized with diamino-3,3′-benzidine substrate. Three to five rinses were performed between each step.

Pictures were then taken from sections containing the locus coeruleus and the nucleus subcoeruleus ventralis using a Leica DFC40 camera connected to an Olympus BH2 microscope. The number of DBH-immunoreactive cells within the boundaries of these nuclei was counted on both sides of the brain in all sections containing these nuclei. Given the small size of these nuclei (150–200 µm length in the rostro-caudal axis) compared to the section thickness (50 µm), they were in all subjects present in only one or two sections. The average number of DBH-immunoreactive cells per section was then computed for each subject and each nucleus and used for statistical analysis. No attempt was made to correct these numbers by the surface or volume of the target nuclei since the boundaries of these nuclei were not visible in immuno-stained sections.

## Supporting Information

Figure S1 **Activations induced by the auditory stimuli (vs. rest) in two individuals (one saline treated bird and one DSP-4 treated bird).** The statistical parametric maps (unilateral one sample t-test) are superimposed on anatomical images coming from the zebra finch atlas. They illustrate the bilateral activation of Field L, the equivalent of the mammalian primary auditory cortex, and the (caudally and frontally) adjacent secondary auditory regions. T values are color coded according to the scales displayed on the right side of the figure. Only voxels in which the t-test was found significant (p value <0.05, corrected for multiple comparisons at the whole brain level) are displayed.
(TIF)
