# Supplementary figures and images for: Own Song Selectivity in the Songbird Auditory Pathway: Suppression by Norepinephrine
Source: PLoS One. 2011 May 23;6(5):e20131. doi: 10.1371/journal.pone.0020131 (PMC3100321; doi:10.1371/journal.pone.0020131)

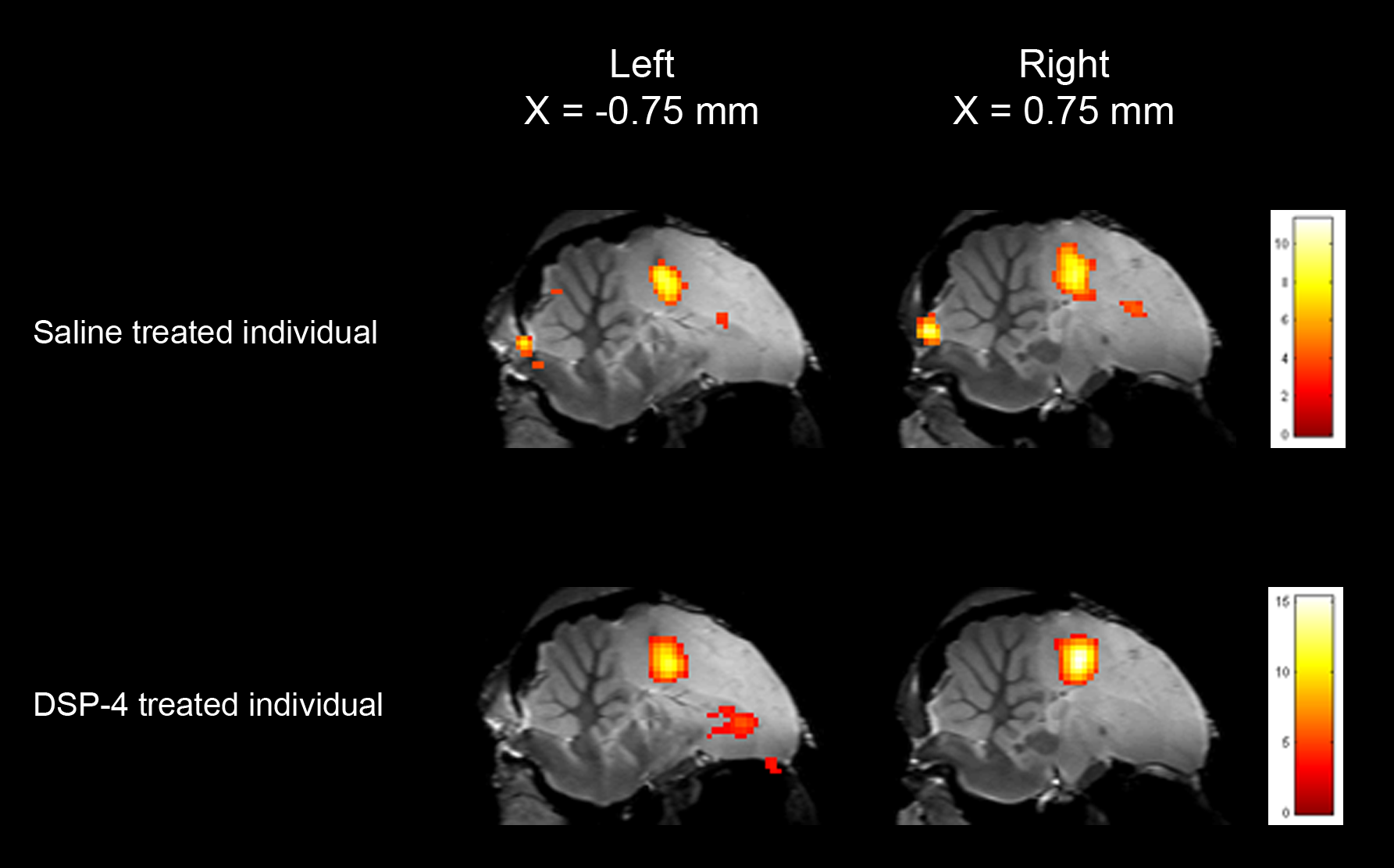

Supplement: Figure S1 — Activations induced by the auditory stimuli (vs. rest) in two individuals (one saline treated bird and one DSP-4 treated bird). The statistical parametric maps (unilateral one sample t-test) are superimposed on anatomical images coming from the zebra finch atlas. They illustrate the bilateral activation of Field L, the equivalent of the mammalian primary auditory cortex, and the (caudally and frontally) adjacent secondary auditory regions. T values are color coded according to the scales displayed on the right side of the figure. Only voxels in which the t-test was found significant (p value <0.05, corrected for multiple comparisons at the whole brain level) are displayed. (TIF) [file pone.0020131.s001.tif]
